# Comparison of Anionic, Cationic and Nonionic Surfactants as Dispersing Agents for Graphene Based on the Fluorescence of Riboflavin

**DOI:** 10.3390/nano7110403

**Published:** 2017-11-22

**Authors:** Rocío Mateos, Soledad Vera, Mercedes Valiente, Ana María Díez-Pascual, María Paz San Andrés

**Affiliations:** 1 Department of Analytical Chemistry, Physical Chemistry and Chemical Engineering, Faculty of Biology, Environmental Sciences and Chemistry, Alcalá University, Alcalá de Henares, 28871 Madrid, Spain; rocio.mateosm@edu.uah.es (R.M.); soledad.vera@uah.es (S.V.); mercedes.valiente@uah.es (M.V.); 2 Institute of Chemistry Research “Andrés M. del Río” (IQAR), University of Alcalá, Ctra, Madrid-Barcelona, Km. 33.6, Alcalá de Henares, 28871 Madrid, Spain

**Keywords:** graphene, surfactants, riboflavin, fluorescence, sodium docecylsulphate, polyoxiethylen-23-lauryl ether, docecylammonium bromide, hexadecyltrimmethylammonium bromide, quenching

## Abstract

Fluorescence quenching is a valuable tool to gain insight about dynamic changes of fluorophores in complex systems. Graphene (G), a single-layered 2D nanomaterial with unique properties, was dispersed in surfactant aqueous solutions of different nature: non-ionic polyoxyethylene-23-lauryl ether (Brij L23), anionic sodium dodecylsulphate (SDS), and cationic hexadecyltrimethylammonium bromide (CTAB) and dodecyltrimethylammonium bromide (DTAB). The influence of the surfactant type, chain length and concentration, G total concentration and G/surfactant weight ratio on the fluorescence intensity of vitamin B_2_ (riboflavin) was investigated. The quality of the different G dispersions was assessed by scanning and transmission electron microscopies (SEM and TEM). A quenching phenomenon of the fluorescence of riboflavin was found for G dispersions in all the surfactants, which generally becomes stronger with increasing G/surfactant weight ratio. For dispersions in the ionic surfactants, the quenching is more pronounced as the surfactant concentration raises, whilst the non-ionic one remains merely unchanged for the different G/Brij L23 weight ratios. More importantly, results indicate that DTAB solutions are the optimum media for dispersing G sheets, leading to an up to 16-fold drop in the fluorescence intensity. Understanding the mechanism in fluorescence quenching of G dispersions in surfactants could be useful for several optical applications.

## 1. Introduction

In recent years, carbon nanomaterials have been investigated due to their outstanding properties. Graphene (G) is amongst the most exciting nanomaterials owing to its exceptional mechanical, electrochemical, electronic and optical properties [1,2,3,4,5]. Since its discovery in 2004 [3,6] it has been used in many applications in analytical chemistry [7,8], particularly for the design of electrochemical and optical sensors [9,10,11]. These exceptional properties make G an ideal filler for reinforcing polymeric matrices, and the resulting nanocomposites have similar or even better performance than those incorporating carbon nanotubes (CNTs) [12]. However, the development of graphene-based materials faces numerous challenges in terms of dispersion and interfacial interaction, particularly at higher G loadings. G sheets tend to restack due to strong van der Waals attractions, a process that reduces their processability and also compromises properties such as accessible surface area. Given that G is a hydrophobic material, for certain applications it needs to be dispersed in liquids such as organic solvents [13] or water, frequently via ultrasonication with the aid of dispersing agents [14].

Different strategies have been reported to yield homogeneous and stable G dispersions in liquid media, including the use of surfactants of different nature and charge (i.e., cationic, anionic and non-ionic) [15,16,17,18,19,20,21,22], as well as the covalent and non-covalent functionalization with polymers [11]. Different mechanisms in stabilizing graphene dispersions have been reported [23]: ionic surfactants wrap the G flakes, providing an effective charge that results in electrostatic repulsion and hinders re-aggregation of the flakes, whereas non-ionic ones provoke steric and polar interactions that also impede agglomeration. In the work presented herein, we aim to comparatively investigate the effect of micellar solutions of surfactants of different nature: non-ionic polyoxyethylene-23-lauryl ether (Brij L23), anionic sodium dodecylsulphate (SDS), and cationic hexadecyltrimethylammonium bromide (CTAB) and dodecyltrimethylammonium chloride (DTAB) for dispersing G. The fluorescence of an important biomolecule such as riboflavin has been used for testing the quality of the obtained dispersions and studying the interactions of this vitamin with G in the dispersions. 

Riboflavin is a water-soluble vitamin, very stable during thermal processing, storage, and food preparation but susceptible to degradation upon exposure to light [24]. This vitamin is frequently transformed into two coenzymes, flavin mononucleotide (FMN) and flavin adenine dinucleotide (FAD), which are important for enzymatic catalysis. Hence, this vitamin is responsible for maintaining healthy blood cells, helping to boost energy levels, preventing free radical damage, contributing to growth, protecting skin and eye health, and participating in the metabolism of other vitamins, fats, carbohydrates, and proteins; on the whole, it plays a key role in the human diet to sustain the development and normal growth of human tissues [25]. The lack of riboflavin can induce a number of serious side effects such as hematological, cardiovascular, and gastrointestinal illnesses, and can be a threat factor for some cancers. For instance, the increase in its ingestion can prevent breast and uterine cancer [26]. Therefore, several analytical techniques such as high-performance liquid chromatography (HPLC) [27], chemiluminescence [28], spectroelectrochemical [29], biosensor technology [30], and capillary electrophoresis [31] have been devised for the determination of riboflavin. However, these methods are highly time-consuming, show lack of selectivity and require large amounts of expensive solvents. Conversely, fluorescence methods are being increasingly renowned owing to their straightforwardness, outstanding sensitivity and selectivity, wide range of applicability, non-invasiveness, and rapidity. In addition, the fluorescence can be improved in a micellar medium, using surfactants [32].

Fluorescence quenching is a subject that has received long-standing attention given that it can serve as a valuable source of information in biochemical systems [33]. A number of recent studies have reported that G is an efficient fluorescence quencher of organic dyes [34,35,36,37,38], and it was found that the lower the polarity of the molecules, the more pronounced the quenching effect was due to stronger interactions [35], which can occur via covalent bonding, although generally it take place through π-π stacking with the G aromatic double bonds. Recently, the interaction between G, graphene oxide (GO) and CNTs with phthalocyanines has been compared using fluorescence [39], and it was found to be considerably stronger for G compared to the other carbon nanomaterials. The interaction of GO with both fluorescein isothiocyanate (FITC) and adenosine triphosphate (ATP) has also been studied and a fluorescence resonance energy transfer (FRET) mechanism was found, showing a stronger quenching of FITC fluorescence by GO than by ATP; this is attributed to the formation of supramolecular assemblies bound with hydrogen bonding and π-π stacking interactions [40]. GO has also been recently studied as a nanocarrier for important molecules for cancer detection such as oligonucleotide molecular beacons encoded for the detection of the biomarker survivin [41]. 

However, to the best of our knowledge, there is no previous systematic study on the quenching behaviour of vitamin B_2_ fluorescence by nanomaterial dispersions in surfactant solutions. The influence of the following parameters on the fluorescence intensity of the riboflavin will be analyzed in this work: (1) nature, chain length and concentration of surfactant; (2) G concentration and (3) G/surfactant weight ratio. Further, the interactions among riboflavin, G and the surfactant effect will be discussed.

## 2. Results and Discussion

### 2.1. Fluorescence of Riboflavin in Aqueous Solutions of Surfactants of Different Nature

**Figure 1:**
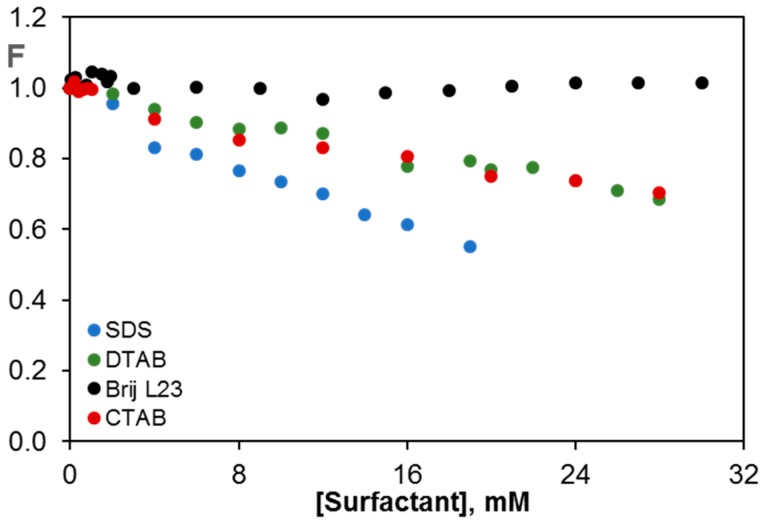
Change in the fluorescence of vitamin B_2_ (F) with increasing concentration of the different surfactants.

To analyze the effect of the surfactant head group on the fluorescence of riboflavin, spectra were recorded in water and in aqueous solutions of SDS, CTAB, DTAB and Brij L23, both above and below their critical micelle concentration (CMC). The 3D fluorescence spectra of the vitamin in water, 10 mM Brij L23, 20 mM SDS and 30 mM DTAB, each of them at a concentration above the CMC, are compared in Figure S1 in the supplementary material. No change in the maximum λ_exc_ or λ_em_ (450 and 518 nm, respectively) is found in the presence of the surfactant solutions either below or above the CMC (see the CMC for each surfactant in Section 3). This indicates that the polarity surrounding riboflavin should be the same and that riboflavin does not solubilize into the micelles. Albeit in the case of the ionic surfactants there is a clear decrease in the fluorescence intensity. This effect can be more clearly visualized in Figure 1 that shows the change in the normalized fluorescence (F) with the concentration of surfactant. 

The non-ionic surfactant Brij L23 hardly affects the fluorescence of the vitamin, which can be explained considering that it is solubilized in water and it does not interact with the non-ionic surfactant. Similar behavior was found for the fluorescence of riboflavin in aqueous polymeric solutions such as polyethylene glycol (PEG) and polaxamer 407 [42,43], attributed to the very weak polymer-vitamin interaction that did not modify the riboflavin fluorescence. Nevertheless, the ionic surfactants cause a decrease in the fluorescence intensity of the vitamin, indicative of the interaction between the riboflavin molecule and the surfactant molecule. This quenching effect is stronger for the anionic surfactant, likely due to the larger space between the head ionic groups of SDS micelles compared to those of CTAB or DTAB, which makes the penetration of the riboflavin molecule into the micelles easier [32]. These results significantly differ from those found for the fluorescence of other aromatic molecules like fluorene in the presence of surfactant solutions [44], where the fluorescence intensity increased in the presence of Brij L23 or CTAB, ascribed to a solubilization effect of fluorene. On the other hand, the comparison of the results obtained for the cationic surfactants CTAB and DTAB, which have identical structure and only differ in the chain length (16 and 12 carbon atoms, respectively) indicates that both have the same effect on the fluorescence of riboflavin. Therefore, it can be concluded that the headgroup nature of the surfactant is the key factor determining the quenching effect observed.

### 2.2. Transmission Electron Microscopy (TEM) Analysis of the G Dispersions 

**Figure 2:**
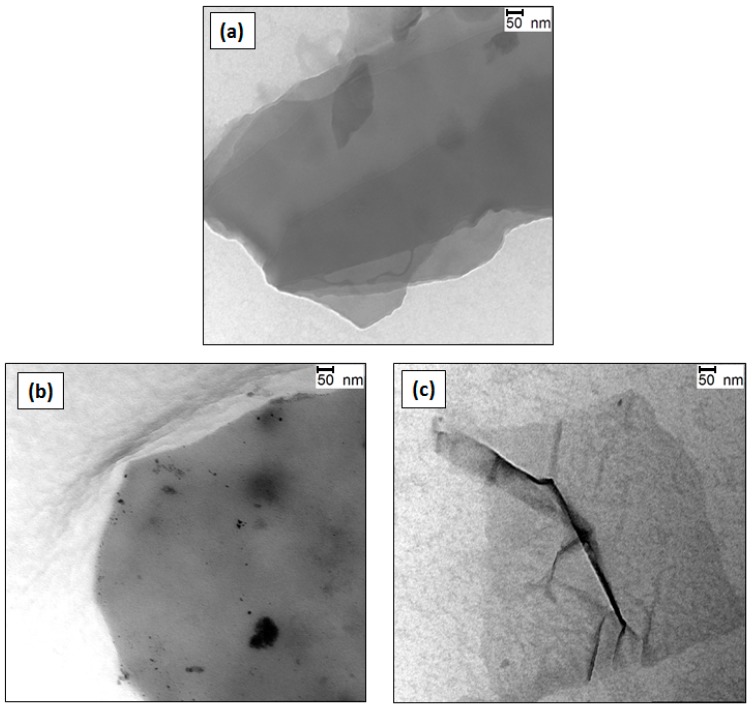
TEM images of G dispersions in 20 mM SDS with G/SDS weight ratios of 0.5 (**a**), 1.0 (**b**) and 2.0 (**c**) wt %.

The quality of G dispersions in the different surfactant solutions was assessed by TEM analysis, and typical images of G (0.5, 1.0 and 2.0 wt %) dispersions in 20 mM SDS solutions are shown in Figure 2. The dispersion with 0.5 wt % G (Figure 2a) is composed of rigid and stacked flakes with thickness in the range of 4–6 nm. The sheets are highly covered by the surfactant and display a soapy aspect. The dispersion with 1.0 wt % G (Figure 2b) also comprises stiff and smooth sheets, albeit these are less wrapped by SDS. Further, small black dots can be observed in the image that could arise from aggregated SDS molecules. In contrast, the dispersion with 2.0 wt % G (Figure 2c) shows very thin and wrinkled G sheets, with a flake thickness <2 nm. These results confirm the exfoliation of G after the ultrasonication in SDS aqueous solutions, and indicate that the degree of exfoliation depends critically on the G/surfactant weight ratio. The G layers coated by SDS are stabilized against re-aggregation by the repulsive electrostatic interactions between nearby SDS-coated flakes, and the concentration of free G, dispersed in the solution, increases linearly with an increasing repulsive electrostatic potential barrier [17], that is, with increasing the number of negative charges onto the G sheets.

**Figure 3:**
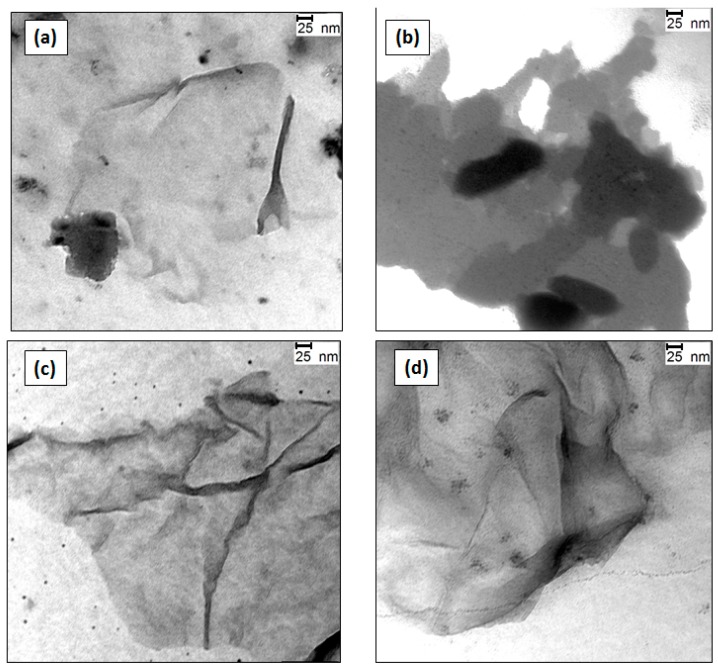
Comparison of TEM images of G (2.0 wt%) dispersions in 20 mM SDS (**a**), 30 mM CTAB (**b**) and DTAB (**c**) as well as 10 mM Brij L23 (**d**).

Representative TEM images of G (2.0 wt %) dispersions in 20 mM SDS, 30 mM CTAB and DTAB as well as 10 mM Brij L23 are compared in Figure 3. A decrease in the flake thickness is found upon dispersion in the surfactant solutions, particularly in the case of DTAB and Brij L23 (Figure 3c,d, respectively), which show skinny and highly disentangled flakes, with an average thickness ≤1 nm, corresponding to monolayer or few-layered graphene. In contrast, the CTAB dispersion (Figure 3b) exhibits a foamy appearance, with thick sheets (in the range of 5–8 nm) well covered by the surfactant. From these images, it can be concluded that DTAB and Brij are more effective to yield well exfoliated and stable G dispersions after the ultrasonication process than CTAB or SDS. This is surprising, given that, whilst for the ionic surfactants the stabilization mechanism is based on electrostatic interactions, for the non-ionic ones it is based on steric and polar effects [17]. Accordingly, the hydrophobic chains of Brij L23 probably adsorb onto the G surface by means of van der Waals and hydrophobic interactions, while the bulky hydrophilic part probably expands into the aqueous solution. When two surfactant-coated sheets become closer to each other, the hydrophilic groups begin to interact, causing an osmotic repulsion between the G flakes. Further, G-wrapped layers have been reported to show a negative zeta potential that contributes to the stabilization of the flakes in the solution [45].

### 2.3. Fluorescence of Riboflavin in G Dispersions in Surfactants

Three different G/surfactant weight ratios were investigated: 0.5, 1.0 and 2.0%, and two different sets of measurement were carried out. The first series were prepared by diluting the original G dispersion with water, in order to obtain different concentrations of surfactant and G whilst keeping the G/surfactant weight ratio (w_G_/w_S_) constant. The second series was prepared by diluting the dispersion with the initial surfactant concentration, hence both the G concentration and G/surfactant weight ratio change whereas the surfactant concentration remains invariable.

#### 2.3.1. Fluorescence of Riboflavin in G Dispersions in Brij L23 Aqueous Solutions

**Figure 4:**
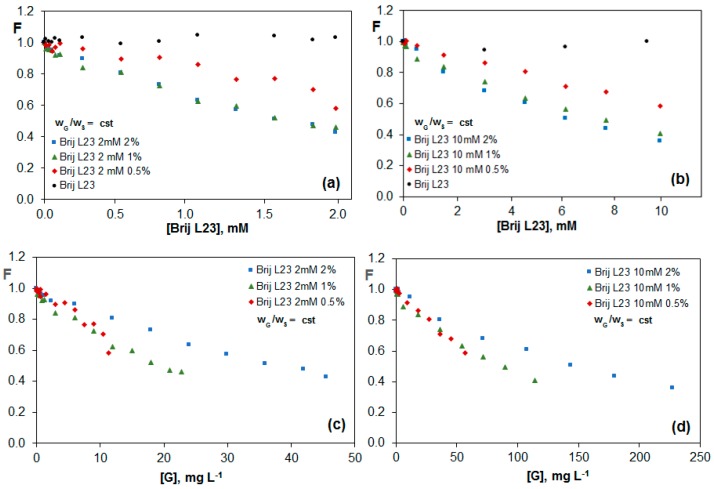
Change in the fluorescence of vitamin B_2_ (F) for G dispersions in 2 mM (**a**,**c**) and 10 mM (**b**,**d**) Brij L23 as a function of surfactant concentration (**a**,**b**) or graphene concentration (**c**,**d**) for solutions with a constant G/surfactant weight ratio.

Figure 4 shows the variation of the fluorescence of riboflavin in the presence of G dispersions in Brij L23 with two different concentrations, 2 and 10 mM, for G/surfactant weight ratios of 0.5, 1.0 and 2.0%. As can be observed in Figure 4a,b, there is a quenching effect of the vitamin fluorescence for all surfactant concentrations that was not observed in the absence of G. This effect increases with increasing G percentage, and is a result of the π-π interactions between the electron-rich aromatic isoalloxazine rings of riboflavin and the electron-acceptor rings of G dispersed in the solution.

The dispersions with 1.0 and 2.0 wt % G almost lead to the same effect, indicative of a saturation behaviour, hence higher percentage of G would not result in a stronger quenching. Interestingly, for each G/surfactant weight ratio, the minimum F value obtained is roughly the same for the two surfactant concentrations, suggesting that the quenching depends on the amount of G in the dispersion with respect to the surfactant, although it is independent on the surfactant and G concentration as long as the G/surfactant ratio remains constant. Similar conclusions can be drawn from Figure 4c,d, which show the change in F as a function of the total G concentration in the solution. This indicates that the non-ionic surfactant covers the G sheets, and only the G surface free from surfactant interacts with the vitamin, thereby decreasing its fluorescence. The micelles in the bulk solution likely do not interact with the vitamin, hence the quenching phenomenon depends only on the G/surfactant ratio. 

**Figure 5:**
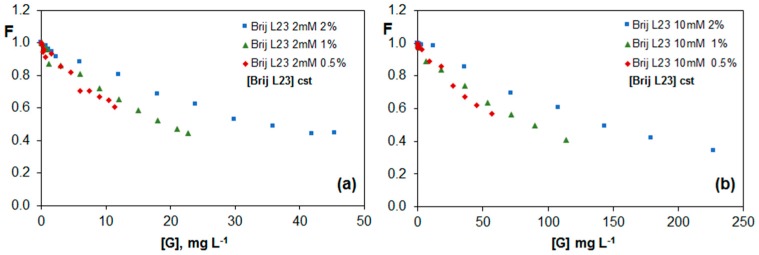
Evolution of fluorescence of vitamin B_2_ (F) vs. G concentration for G dispersions in 2 mM (**a**) and 10 mM (**b**) Brij L23 for solutions with a constant surfactant concentration.

Figure 5a,b show the change in the riboflavin fluorescence as a function of G concentration for G dispersions (0.5, 1.0 and 2.0 wt %) in 2 and 10 mM Brij L23, respectively, maintaining constant the surfactant concentration. The comparison of these figures reveals that the quenching effect is very similar for both surfactant concentrations. Further, the values obtained are very close to those found in Figure 4c,d for constant G/surfactant weight ratios. This corroborates that the main factor influencing the fluorescence of the vitamin is the percentage of G in the dispersion with respect to the surfactant. As we have mentioned, the riboflavin does not interact with the nonionic surfactant in water. In this case, the surfactant likely causes contradictory effects: on the one hand, it facilitates the exfoliation of the G sheets, while on the other hand, it hampers the vitamin-G interaction. Therefore, the free G layers that are not covered by the surfactant depends exclusively on the G/Brij ratio in the dispersion and not on the Brij L23 or G total concentrations.

#### 2.3.2. Fluorescence of Riboflavin in G Dispersions in SDS Aqueous Solutions

**Figure 6:**
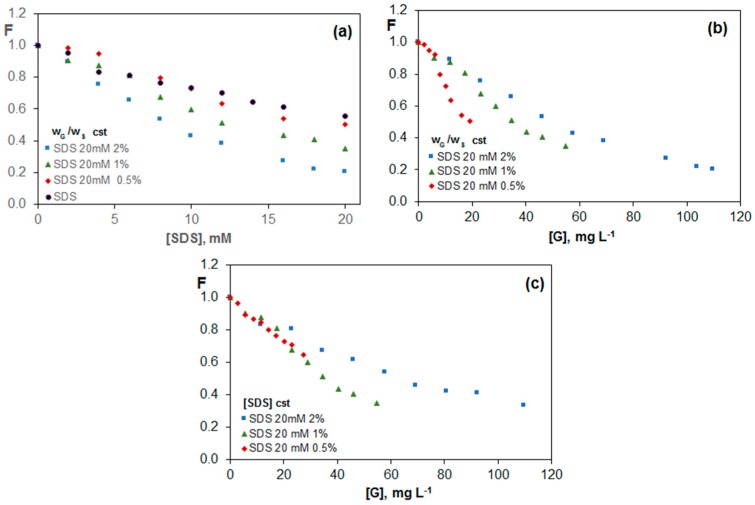
Evolution fluorescence of vitamin B_2_ (F) for G/SDS dispersions as a function of SDS (**a**) and graphene (**b**,**c**) concentration for solutions with a constant G/surfactant weight ratio (**a**,**b**) or constant SDS concentration (**c**).

Figure 6a shows the fluorescence of riboflavin in 20 mM SDS solution and G/SDS dispersions as a function of the surfactant concentration. The quenching effect observed in aqueous solutions of SDS increases in the presence of G, and becomes more pronounced as the G percentage rises. The same conclusion can be drawn from Figure 6b, which shows the change in F vs. G concentration. Therefore, the fluorescence quenching depends on the concentration of both SDS and G, as well as on the G/SDS weight ratio, and is likely due to the presence of the surfactant either in the form of monomers or micelles that interact with the riboflavin molecule. Interestingly, a change in the slope of the plot F vs. SDS concentration is found close to the CMC value, indicative of different interactions riboflavin-monomer and riboflavin-micelle. This is consisted with the results reported by Hsieh et al. [46], who investigated the adsorption of SDS onto functionalized G and found a transition from hemicylindrical micelle structures adsorbed at the G surface to the formation of micelles in the bulk solution at the CMC.

For the three percentages of G studied, the quenching effect is stronger with increasing SDS concentration; this fact indicates that the G sheets covered by the surfactant interact stronger with riboflavin than the free G layers in solution. The change in F with increasing G concentration for a constant SDS concentration of 20 mM and G/SDS weight ratios of 0.5, 1.0 and 2.0 wt % is shown in Figure 6c. In this case, as the surfactant concentration remains constant, it does not modify the fluorescence of riboflavin, and the differences between the three G percentages are due to the total G concentration. For equal G concentration, the decrease in F for dispersions with 0.5 and 1.0% G is almost the same. However, the maximum drop attained in the dispersion with 2.0 wt % G, corresponding to a G concentration of 110 mg L^−1^ is very similar to that obtained for the dispersion with 1.0 wt % that has half of the total G concentration. This behaviour is consistent with the observations from TEM images, which reveal that the dispersions with lower G percentages are significantly more covered by the surfactant, with a soapy aspect.

#### 2.3.3. Fluorescence of Riboflavin in G Dispersions in DTAB and CTAB Aqueous Solutions

**Figure 7:**
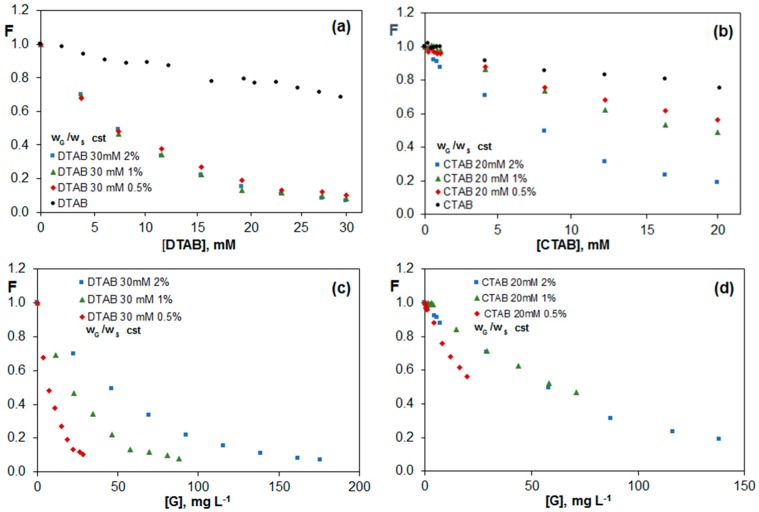
Fluorescence of vitamin B_2_ (F) vs. surfactant (**a**,**b**) or graphene (**c**,**d**) concentration for G dispersions in 30 mM DTAB (**a**,**c**) and 20 mM CTAB (**b**,**d**), for solutions with a constant G/surfactant weight ratio.

In order to investigate the effect of the surfactant chain length, two surfactants with the same head group (trimethylammonium) but with different chain length (12 and 16 carbon atoms, DTAB and CTAB, respectively) were chosen. The comparison of Figure 7a,b reveals that although in the absence of G, the variation of riboflavin fluorescence is the same for both surfactants, in the presence of a G dispersion in the surfactants, the behavior is completely different, and DTAB with a shorter chain length leads to a more prominent quenching, suggesting that it provides a dispersion with more free G sheets able to interact with the vitamin. This is consistent with TEM images (Figure 3b,c), which revealed that the G sheets are more covered by the surfactant in CTAB dispersions; further, in DTAB, the amount of material exfoliated is higher than in CTAB. This difference explains the stronger quenching effect found for DTAB dispersions. It should be noticed that the quenching efficiency obtained for this surfactant is very high, close to 90%. To the best of our knowledge, such high quenching efficiencies with G have only been previously reported for proteins containing aromatic rings [34]. Surprisingly, for this surfactant, the effect of the G sheets over riboflavin fluorescence is the same for the three percentages studied (Figure 7a) while in the case of CTAB, the higher the G percentage, the stronger the quenching effect (Figure 7b). 

A potential explanation for this behavior could be that the DTAB monomer with the shorter chain than CTAB can be adsorbed onto G sheets in larger proportions than CTAB. In addition, since the CMC of DTAB is more than 15 times higher than that of CTAB, the concentration of monomers in these dispersions should be very high and roughly identical for the different percentages, hence the quenching effect is the same independently of the amount of nanomaterial. Therefore, it seems that DTAB monomers are responsible for the quenching effect, and the fluorescence intensity hardly changes at concentrations above the CMC value. Further, the fact that the quenching is very similar for the three G percentages studied is likely due to the fact that all DTAB monomers are adsorbed on the G surface as a monolayer. However, in the case of CTAB dispersions, since the CMC is much lower, the number of monomers should be very low, being that the micelles are responsible for the quenching phenomenon, and the higher the number of micelles, the stronger the quenching. For the three ionic surfactants investigated, we have found that the quenching becomes stronger with increasing surfactant concentration, whilst the non-ionic one remains merely unchanged, in agreement with previous works [23] that revealed a growth in the final graphene concentration in the solution as the concentration of the ionic surfactants augmented, whereas for the non-ionic ones it was roughly maintained.

Figure 7c,d show the change in F vs. G concentration for the two surfactants. For DTAB dispersions, the minimum F value attained is again the same for the three percentages studied, although in the dispersion with lower percentage, the CMC value is reached with a lower total G concentration, which is consistent with the abovementioned fact that the monomers induce the decrease of the fluorescence of riboflavin. However, regarding CTAB dispersions, the minimum F value obtained diminishes with increasing G percentage, given that the micelles induce the quenching effect. 

**Figure 8:**
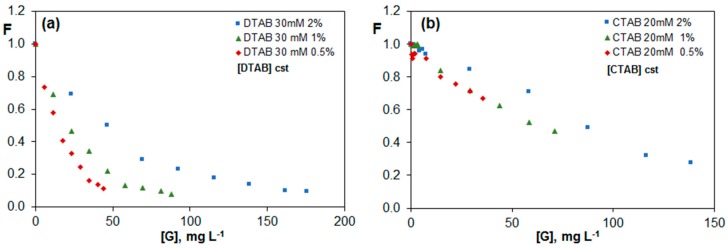
Fluorescence of vitamin B_2_ (F) vs. G concentration for G dispersions in 30 mM DTAB (**a**) and 20 mM CTAB (**b**) for solutions with a constant surfactant concentration.

Figure 8a,b show the evolution of F with G concentration for DTAB and CTAB, respectively, similar to Figure 7, albeit maintaining constant the surfactant concentration. In the case of DTAB (Figure 8a), F values for the three G percentages are almost identical to those in Figure 7c for a constant G/DTAB weight ratio, corroborating that the quenching is only dependent on the concentration of monomers. Regarding CTAB (Figure 8b), the tendency is the same for dispersions with 0.5 and 1.0 wt % G, while for dispersions with a constant G/CTAB weight ratio (Figure 7d) the trend was similar for those with 1.0 and 2.0 wt %. Therefore, for this cationic surfactant, the behaviour is not as clear as for DTAB, and both the micelles and monomers in equilibrium are responsible for the quenching. 

#### 2.3.4. Comparison of the Quenching Effect in G (2 wt %) Dispersions in the Different Surfactants 

**Figure 9:**
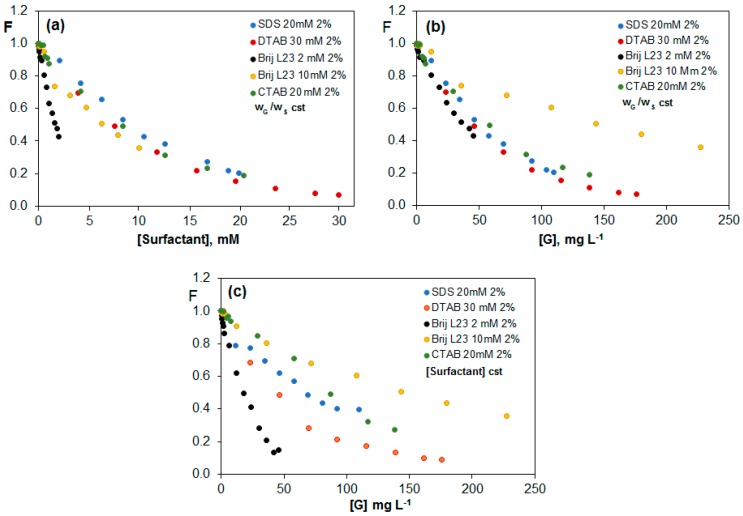
Comparison of the quenching effect for G (2 wt %) dispersions in the different surfactants as a function of surfactant (**a**) and G (**b**,**c**) concentration, for dispersions with a constant G/surfactant weight ratio (**a**,**b**) or constant surfactant concentration (**c**).

Figure 9 compares the change in F with the surfactant (a) and G (b) concentration for G (2 wt %) dispersions in the different surfactants, maintaining the G/surfactant ratio constant. The values have been normalized to the fluorescence of riboflavin in each surfactant (F_0_) in the absence of G. The decrease in fluorescence caused by G dispersions in the three ionic surfactants (SDS, CTAB and DTAB) follows a similar trend (Figure 9a), leading in the three cases to a very high quenching efficiency for a surfactant concentration of approximately 20 mM (i.e., in the range of 80–85%). More importantly, an unprecedented 16-fold drop in the fluorescence of riboflavin is found for the dispersion in DTAB when the maximum G concentration is reached (176 mg L^−1^). This drop is higher than that found in the presence of G (2 wt %) dispersions in (PEG) and polaxamer 407 [42,43], corroborating the effectiveness of this surfactant for attaining well dispersed G dispersions. Further, it is also considerably stronger than that reported for the quenching of different aromatic compounds like phthalocyanines [39] or aromatic dyes [47] in the presence of G dispersions. In contrast, the non-ionic surfactant shows a different trend, with a faster drop in F with increasing surfactant concentration for dispersions prepared in 2 mM Brij L23 (Figure 9a), while leading to higher F values than the ionic surfactants when plotted vs. G concentration for dispersions prepared in 10 mM Brij L23 (Figure 9b). When the surfactant concentration remains constant (Figure 9c), the dispersion in 2 mM Brij L23 shows a stronger decrease at low G concentrations, probably due to the lower coverage of the G sheets in comparison to those in 10 mM Brij L23. Given that the riboflavin molecule does not interact with Brij L23 in aqueous solution, the free G sheets are responsible for the marked quenching found at low G concentrations. This behaviour is consistent with the observations from SEM analysis (Figure S2), which revealed that G sheets are much thinner in 2 mM Brij L23 dispersions than in 10 mM Brij L23, which exhibit a clear soapy aspect.

It should also be noted that the decrease in F for dispersions in 2 mM Brij L23 at low G concentrations is the strongest considering all the surfactants. It is possible that in this medium the G sheets are less covered and interact more easily with the vitamin.

Table 1 summarizes the F/F_0_ ratios along with their standard deviation obtained for riboflavin in four independent G/surfactant dispersions (F) and in the surfactant solutions (F_0_) studied. F_0_ values correspond to the fluorescence of riboflavin in 2 mM Brij L23, 10 mM Brij L23, 20 mM CTAB and 30 mM DTAB, respectively. The ratio between the fluorescence of riboflavin in the surfactant solution (F_0_) and the fluorescence in water (F_W_) has been included because ionic surfactants produce fluorescence quenching in the absence of G. For all the surfactants, a decrease in F/F_0_ is found with increasing G percentage. Regarding the influence of the surfactant concentration, the decrease in F/F_0_ is slightly more pronounced for the dispersions prepared in 10 mM Brij L23 compared to those prepared in 2 mM. Further, the drop-in intensity is somewhat stronger for the dispersions with higher G percentages. Therefore, it can be concluded that the fluorescence decreases only due to the presence of G, and the drop is only dependent on the G concentration and G/Brij L23 weight ratio.

For SDS dispersions, a decrease in F/F_0_ is also found, and the value obtained for a G (2 wt %) dispersion is lower than those of Brij L23 dispersions. In the case of cationic surfactants, the diminution in F/F_0_ ratio with G percentage is stronger for DTAB than for CTAB. The cationic surfactant DTAB produces the highest quenching effect in the riboflavin fluorescence, even with lower G percentages in the dispersion. For DTAB dispersions, the fluorescence of riboflavin slightly decreases with increasing G percentage, and the standard deviations are very low, while for CTAB, the values show higher variability and thus, lower reproducibility. Overall, taking into account fluorescence data and TEM analysis, it can be concluded that DTAB is the best medium for dispersing the G sheets.

#### 2.3.5. Study of the Quenching Process

The diminution in the fluorescence intensity of a fluorophore, that is, the quenching phenomenon, can occur primarily by two processes: electron transfer or energy transfer [48]. In both cases, the excited state energy of the fluorophore is deactivated due to the presence of the quencher. There are two scenarios by which quenching is generally modelled as shown below. When the quenching takes place by the quencher diffusing through the solution and interacting with the fluorophore, resulting in another deactivation pathway in competition with the fluorescence, it is named as dynamic quenching. This type of quenching is controlled by how fast the quencher can diffuse through the solution and “collide” with the fluorophore, and it is usually a very fast and efficient process. The dynamic quenching shows a concentration-dependence that can be described by the Stern-Volmer equation:

F_0_/F = 1 + k_q_τ_0_[Q] = 1 + K_SV_[Q] (1)

where F_0_ and F are the fluorescence in the absence and in the presence of the quencher, K_SV_ is the Stern-Volmer constant, [Q] is the concentration of quencher, k_q_ is the quenching rate constant, and τ_0_ is the natural radiative lifetime of the fluorophore in the absence of quencher.

However, if the quencher is somehow associated with the fluorophore in solution prior to light absorption, i.e., via formation of a ground-state complex, the fluorophore will not emit, due to induced changes in its properties because of presence of quencher. Therefore, the reduction in emission intensity will be affected by the extent to which the quencher associates to the fluorophore and the number of quenchers present. This type of quenching is described by another linear relationship replacing K_SV_ by K_S_, the equilibrium constant of association between the fluorophore and the quencher:

F_0_/F = 1 + K_S_[Q] (2)

Deviations from the linear plot take place when both types of quenching occur simultaneously. Such combined quenching appears as upward curvature, and can be described by a second order polynomial.

To obtain information about the quenching mechanism in the investigated systems, the ratio of the fluorescence intensity in the absence and the presence of G dispersions in the different surfactants was plotted as a function of both surfactant and G concentration (for the dispersions with constant G/surfactant weight ratio) and versus G concentration (for the dispersions with a constant surfactant concentration), and the results are shown in Figures S3–S7. For the four surfactants investigated, a linear behaviour is found at low G concentrations, which deviates from the linearity at high nanomaterial contents, showing an upward curvature indicative of the combination of two or more quenching mechanisms. For high G concentrations, the inner filter effect can produce a deviation of the linearity as it was observed previously for GO nanoplatelets [41]. This fact occurs when a high concentration of G sheets provokes a light scattering that limits the fluorescence measurements as a result of the optical density of the solutions. However, G concentrations in this work are relatively low; in fact, F_0_/F ratio is linear up to a G concentration of 230 mg L^−1^ for the dispersions in Brij L23, which show the highest G loadings. 

Regarding G dispersions in SDS (Figure S3), a change in the slope of the curve F_0_/F vs. surfactant concentration is found for the three G percentages studied in the vicinity of the CMC, suggesting a change in the quenching mechanism when micelles start to develop in the medium. When F_0_/F is plotted vs. G concentration, the change in the slope appears at concentrations of about 10, 20 and 40 mg L^−1^ for G percentages of 0.5, 1.0 and 2.0 wt %, respectively. 

In the case of dispersions in Brij L23 (Figures S4–S5), a linear relationship is found for both surfactant concentrations studied, 2 and 10 mM, and the dispersions in the highest Brij L23 concentration show better reproducibility. It is worthy to note that, for both concentrations, the trend of the plot F_0_/F vs. G and the values obtained are very similar, despite the fact that the concentrations of G are much higher when the surfactant concentration is 10 mM. This confirms the dependence of F_0_/F with the G/surfactant weight percentage. 

With regard to the cationic surfactants, a clear deviation from the linearity is found in the plot F_0_/F vs. CTAB concentration (Figure S6) for the G/surfactant weight ratio of 2%, although the deviation does not match with the CMC, but occurs at about 10 times the CMC value. Nevertheless, the quenching caused by G dispersed in CTAB is slightly significant compared to that induced by G dispersions in DTAB (Figure S7). In this case, a clear change in slope is found at a surfactant concentration of 14 mM that corresponds to its CMC. Moreover, the plots are almost identical for dispersions with G/surfactant weight ratio constant and surfactant concentration constant, which corroborates the great potential of DTAB to disperse G in an effective and reproducible way.

Table 2 shows the quenching constants (K) obtained from the plots F_0_/F as a function of G concentration (Figures S3–S7) irrespective of the type of quenching that occurs. 

The values have been calculated both for the solutions with a constant G/surfactant weight ratio and for solutions with a constant surfactant concentration in the concentration range where the data follow a straight line with intercept 1. As can be observed in this table, the highest values are obtained for DTAB dispersions, in particular for those with 0.5 wt % G, maintaining constant either the G/DTAB weight ratio or the DTAB concentration. Regarding CTAB dispersions, K values are also higher for those with 0.5 wt % G, albeit the values are significantly lower than those in DTAB.

On the other hand, dispersions in 2 mM Brij L23 show higher values than those in 10 mM. This fact confirms that the increase in the surfactant concentration increases the degree of covering of the G sheets and the quenching over riboflavin fluorescence decreases. Therefore, the lower the surfactant concentration, the stronger is the quenching produced over the fluorescence of riboflavin. Further, it is more pronounced for the cationic surfactant with a shorter chain. The values obtained for solutions prepared maintaining the G/surfactant ratio or the surfactant concentration constant do not show significant differences, and the quenching depends on the quality of the G dispersion in the surfactant.

## 3. Materials and Methods 

### 3.1. Reagents 

All the reagents were of analytical grade and were used without further purification. AvanGRAPHENE, G powder with lamellar structural morphology comprising less than 6 layers with a thickness ≤2 nm, was supplied by Avanzare Innovación Tecnológica, SL (Logroño, Spain). Riboflavin (C_17_H_20_N_4_O_6_, Mw = 376.36 g mol^−1^), sodium dodecylsulphate (SDS, NaC_12_H_25_SO_4_, micellar critical concentration CMC = 8.2 mM, Mw = 288.38 g mol^−1^), dodecyltrimethylammonium chloride (DTAB, CH_3_(CH_2_)_11_N(CH_3_)_3_Br, CMC = 14.0 mM, Mw = 308.34 g mol^−1^) and polyoxyethylene-23-lauryl ether (Brij L23, C_12_H_25_(OCH_2_CH_2_)_23_OH, CMC = 91 μM, Mw = 1198.56 g mol^−1^), were purchased from Sigma–Aldrich (Madrid, Spain). Hexadecyltrimethylammonium bromide (CTAB, C_19_H_42_BrN, Mw = 364.46 g mol^−1^, CMC = 0.9 mM) was obtained from Merck (Barcelona, Spain). All the aqueous solutions were prepared using ultrapure water obtained from a Milli-Q system (Millipore, Milford, CT, USA).

### 3.2. Instrumentation

Scanning electron microscopy (SEM) measurements were performed using a Hitachi TM-1000 Tabletop microscope (Hitachi High-Technologies, Tokyo, Japan). The G dispersions were dried for a few days and then covered with a ~50 nm Au layer. The solid state samples were observed at different magnifications by means of an electron beam accelerated at 15 kV, under high vacuum. The dispersions were also observed by transmission electron microscopy (TEM) using a Zeiss EM-10C/CR instrument (Oberkochen, Germany) operating at a voltage of 60 kV.

Fluorescence spectra were recorded at 25 °C with a PerkinElmer LS-50B luminescence spectrophotometer equipped with a Xe flash lamp and quartz cuvettes of 1 cm path length thermostatised with a Thermomix BU bath. The excitation and emission slit widths were 5 nm and scan speed 1000 nm min^−1^. The acquisition and data analysis were performed using the Perkin-Elmer FLwin Lab software.

Solutions were degassed using an ultrasonic bath (Selecta, Barcelona, Spain). A Hielscher UP400S ultrasonic tip (Hielscher Ultrasonics GmbH, Teltow, Germany) equipped with a titanium sonotrode with a diameter of 7 mm and length of 100 mm was used to prepare the G dispersions. Dispersions were centrifuged using an Orto Alresa Digicen refrigerated centrifuge (Madrid, Spain).

### 3.3. Procedure

#### 3.3.1. Preparation of the Vitamin Solutions and the Graphene Dispersions in the Surfactant Solutions

A stock solution of riboflavin (250 mg L^−1^) was prepared in ultrapure water and stored at 4 °C in glass beakers under dark conditions. Working solutions were prepared by dilution and the final riboflavin concentration for the different fluorescence measurements was 0.6 mg L^−1^. The G dispersions in the different surfactant solutions were prepared by weighing the proper amount of both components and diluting them with ultrapure water. Subsequently, the solutions were placed in the ultrasonic bath for 1 h followed by sonication with the ultrasonic probe for 5 min at a power of 160 W and frequency of 24 kHz. Finally, they were centrifuged at 4000 rpm for 1 h and stored in amber glass beakers. All the dispersions were prepared at least in duplicate to check for repeatability.

#### 3.3.2. Riboflavin Fluorescence Spectra in the Different Media

The fluorescence spectra of the vitamin in the presence of increasing concentration of the surfactants, both above and below their CMC, were recorded at T = 25.0 ± 0.1 °C. The initial excitation wavelength (λ_exc_) was set at 280 nm, and 25 spectra were registered with a λ increment of 10 nm to attain the 3D spectra that were projected in two dimensions to find out the optimal λ_exc_ and λ_em_ as well as the corresponding fluorescence intensities. Similarly, the spectra of riboflavin in the G dispersions in the different surfactants were obtained. Two sets of measurements were carried out: one by varying the G concentration (hence the G/surfactant weight ratio) although keeping the surfactant concentration constant, and the other by modifying the surfactant and G concentrations although maintaining the G/surfactant weight ratio. For each surfactant, three G weight ratios were prepared: 0.5, 1.0 and 2.0 wt %. 

## 4. Conclusions

A systematic comparative study of the quenching effect of surfactants with different head group (non-ionic Brij L23, anionic SDS and cationic DTAB) and chain length (cationic DTAB and CTAB) as well as G dispersions in these surfactants on the fluorescence intensity of riboflavin has been carried out. 

Regarding to the surfactant solutions, the non–ionic surfactant hardly affects the fluorescence of the vitamin, whilst a drop in intensity is found for the ionic surfactants, ascribed to stronger vitamin-surfactant interactions. Nevertheless, the vitamin is not solubilized into the micelles and the interactions take place with the ionic headgroups of the surfactant monomers and/or the surface charge of the micelles. Therefore, no effect has been found by changing DTAB to CTAB.

In contrast, a remarkable quenching phenomenon has been observed for G dispersions in all the surfactants, both nonionic and ionic, given that all of them act as dispersing agents, thus promoting the exfoliation of G flakes in the solutions. Regarding dispersions in the ionic surfactants, the quenching becomes stronger with increasing G/surfactant weight ratio and surfactant concentration. The hydrophobic part of the surfactant now plays an important role. The strongest quenching effect is found for the cationic surfactant with shorter chain length (DTAB), which leads to the most effective dispersing mechanism, as revealed by SEM and TEM analyses. The quenching effect observed for G dispersions in the nonionic surfactant (Brij L23) remains merely unchanged with increasing surfactant concentrations as the G surface free from the surfactant interacts with the vitamin. This work paves the way towards a better choice of dispersing agent for developing nanomaterial based systems for the determination of compounds of biological interest.

## Figures and Tables

**Table 1:** Fluorescence ratios of riboflavin in the G dispersions and in the surfactant solutions. (F/F_0_: ratio of the fluorescence in G dispersions in the surfactant solutions (F) to the fluorescence in the surfactant solutions (F_0_); F_0_/F_W_: ratio of the fluorescence in the surfactant solutions to the fluorescence in water, F_W_).

		**Brij L23 2 mM**		**Brij L23 10 mM**
**G, wt %**	**F_0_/F_W_**	**F/F_0_**	**F_0_/F_W_**	**F/F_0_**
0	1.04		1.00	
0.5		0.62 ± 0.03		0.58 ± 0.01
1.0		0.46 ± 0.01		0.41 ± 0.01
2.0		0.42 ± 0.03		0.36 ± 0.01
		**SDS 20 mM**		
**G, wt %**	**F_0_/F_W_**	**F/F_0_ [SDS] = cte**		
0	0.55			
0.5		0.6 ± 0.1		
1.0		0.36 ± 0.03		
2.0		0.28 ± 0.06		
		**DTAB 30 mM**		**CTAB 20 mM**
**G, wt %**	**F_0_/F_W_**	**F/F_0_**	**F_0_/F_W_**	**F/F_0_**
0	0.68		0.75	
0.5		0.10 ± 0.01		0.61 ±0.06
1.0		0.08 ± 0.01		0.49 ± 0.02
2.0		0.08 ± 0.01		0.23 ± 0.04

**Table 2:** Quenching constants (K) obtained from the plots F_0_/F vs. G concentration for G (0.5, 1 and 2 wt %) dispersions in the different surfactants in the linear concentration range with intercept 1.

DISPERSION	[Surfactant], mM	G, mg L^−1^	K × 10^3^, L mg^−1^	K, L mg^−1^
SDS 20 mM	0–20	0	0.135 ± 0.003	–––
SDS 20 mM/G 0.5%	0–5	0–10	G/SDS wt % constant	0.018 ± 0.006
SDS 20 mM/G 1%	0–6	0–20		0.02 ± 0.01
SDS 20 mM/G 2%	0–8	0–35		0.021 ± 0.003
SDS 20 mM/G 0.5%	20	0–10	[SDS] constant	0.017 ± 0.002
SDS 20 mM/G 1%	20	0–55		0.024 ± 0.005
SDS 20 mM/G 2%	20	0–110		0.018 ± 0.002
Brij L23 2 mM	0–2	0	0	–––
Brij L23 2 mM/G 0.5%	0–2	0–12	G/Brij L23 wt % constant	0.049 ± 0.004
Brij L23 2 mM /G 1%	0–2	0–23		0.048 ± 0.003
Brij L23 2 mM/G 2%	0–2	0–42		0.04 ± 0.02
Brij L23 2 mM/G 0.5%	2	0–12	[Brij L23] constant	0.049 ± 0.004
Brij L23 2 mM/G 1%	2	0–23		0.049 ± 0.002
Brij L23 2 mM/G 2%	2	0–42		0.032 ± 0.002
Brij L23 10 mM	0–10	0	0	–––
Brij L23 10 mM/G 0.5%	0–10	0–60	G/Brij L23 wt % constant	0.012 ± 0.001
Brij L23 10 mM/G 1%	0–10	0–120		0.012 ± 0.001
Brij L23 10 mM/G 2%	0–10	0–230		0.0069 ± 0.0005
Brij L23 10 mM/G 0.5%	10	0–60	[Brij L23] constant	0.0133 ± 0.0008
Brij L23 10 mM/G 1%	10	0–120		0.010 ± 0.001
Brij L23 10 mM/G 2%	10	0–230		0.0080 ± 0.0008
CTAB 20 mM	0–20	0	0.041 ± 0.002	–––
CTAB 20 mM/G 0.5%	0–20	0–35	G/CTAB wt % constant	0.022 ± 0.001
CTAB 20 mM/G 1%	0–20	0–70		0.0148 ± 0.0008
CTAB 20 mM/G 2%	0–8	0–60		0.018 ± 0.003
CTAB 20 mM/G 0.5%	20	0–35	[CTAB] constant	0.0075 ± 0.0009
CTAB 20 mM/G 1%	20	0–70		0.0066 ± 0.0007
CTAB 20 mM/G 2%	20	0–60		0.007 ± 0.001
DTAB 30 mM	0–30	0	0.045 ± 0.006	–––
DTAB 30 mM/G 0.5%	0–12	0–25	G/DTAB wt % constant	0.09 ± 0.03
DTAB 30 mM/G 1%	0–12	0–35		0.06 ± 0.03
DTAB 30 mM/G 2%	0–12	0–92		0.03 ± 0.02
DTAB 30 mM/G 0.5%	30	0–25	[DTAB] constant	0.09 ± 0.03
DTAB 30 mM/G 1%	30	0–35		0.06 ± 0.01
DTAB 30 mM/G 2%	30	0–50		0.02 ± 0.03

## Supplementary Materials

The following are available online at www.mdpi.com/2079-4991/7/11/403/s1.

## Founding

This work was supported by University of Alcalá [Project CCG2015/EXP005] and Ministerio de Economía y Competitividad (MINECO) [Project CTQ2015-66575-P].
