# Supplementary material for: Comparison of Anionic, Cationic and Nonionic Surfactants as Dispersing Agents for Graphene Based on the Fluorescence of Riboflavin
Source: Nanomaterials (Basel). 2017 Nov 22;7(11):403. doi: 10.3390/nano7110403 (PMC5707620; doi:10.3390/nano7110403)
Supplement: Supplementary file 1 [file nanomaterials-07-00403-s001.pdf]

Supporting information

Comparison of Anionic, Cationic and Nonionic Surfactants as Dispersing Agents for Graphene Based on the Fluorescence of Riboflavin

Rocío Mateos [1], Soledad Vera [1], Mercedes Valiente [1,2], Ana María Díez-Pascual [1,2,*] and María Paz San Andrés [1,*]

[1] Department of Analytical Chemistry, Physical Chemistry and Chemical Engineering, Faculty of Biology, Environmental Sciences and Chemistry; 28871, Alcalá de Henares, Madrid, Spain; rocio.mateosm@edu.uah.es (R.M.); soledad.vera@uah.es (S.V.); mercedes.valiente@uah.es (M.V.)

[2] Institute of Chemistry Research "Andrés M. del Río" (IQAR), University of Alcalá, Ctra. Madrid-Barcelona, Km. 33.6, 28871 Alcalá de Henares, Madrid, Spain

* Correspondence: am.diez@uah.es (A.M.D.-P.); mpaz.sanandres@uah.es (M.P.S.); Tel.: +34-918-856-430 (A.M.D.-P.); +34-918-855-095 (M.P.S.)

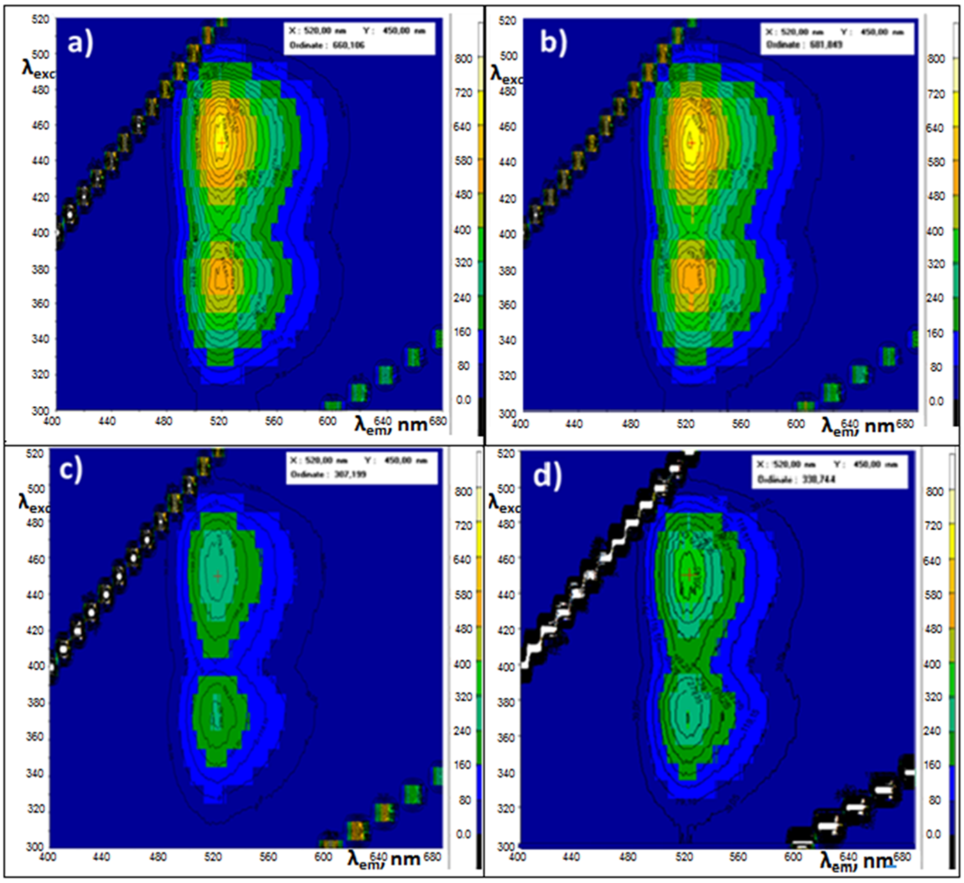

Figure S1. Fluorescence spectra of riboflavin 0.6 mg L^{-1} in a) water, b) 10 mM Brij L23, c) 20 mM SDS and d) 30 mM DTAB.

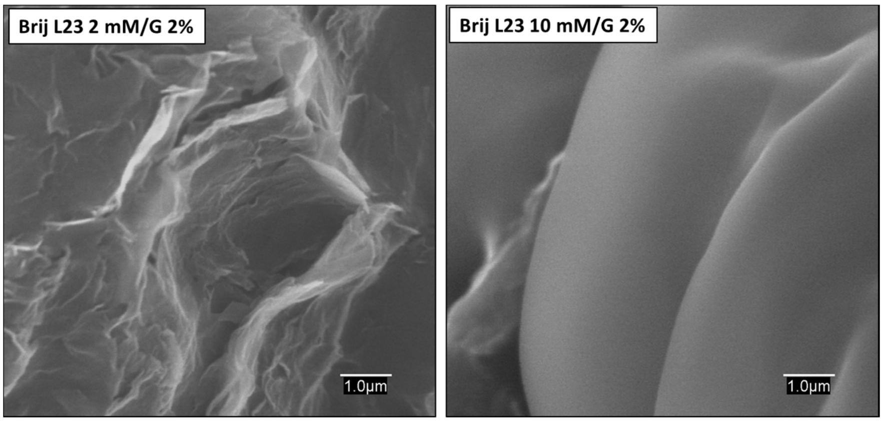

Figure S2. SEM images of G (2 wt%) dispersion in 2 mM Brij L23 (a) and 10 mM Brij L23 (b).

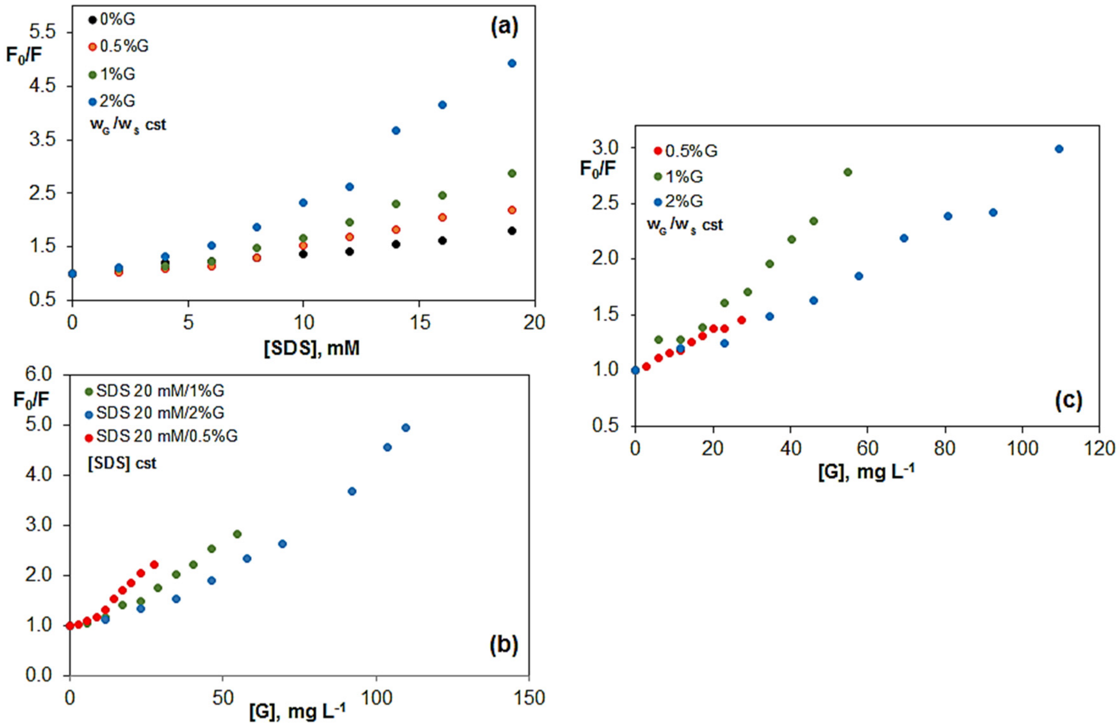

Figure S3. F_0/F for G dispersions in 20 mM SDS as a function of surfactant (a) and G (b and c) concentration, for dispersions with a constant G/surfactant weight ratio (a,b) or constant surfactant concentration (c).

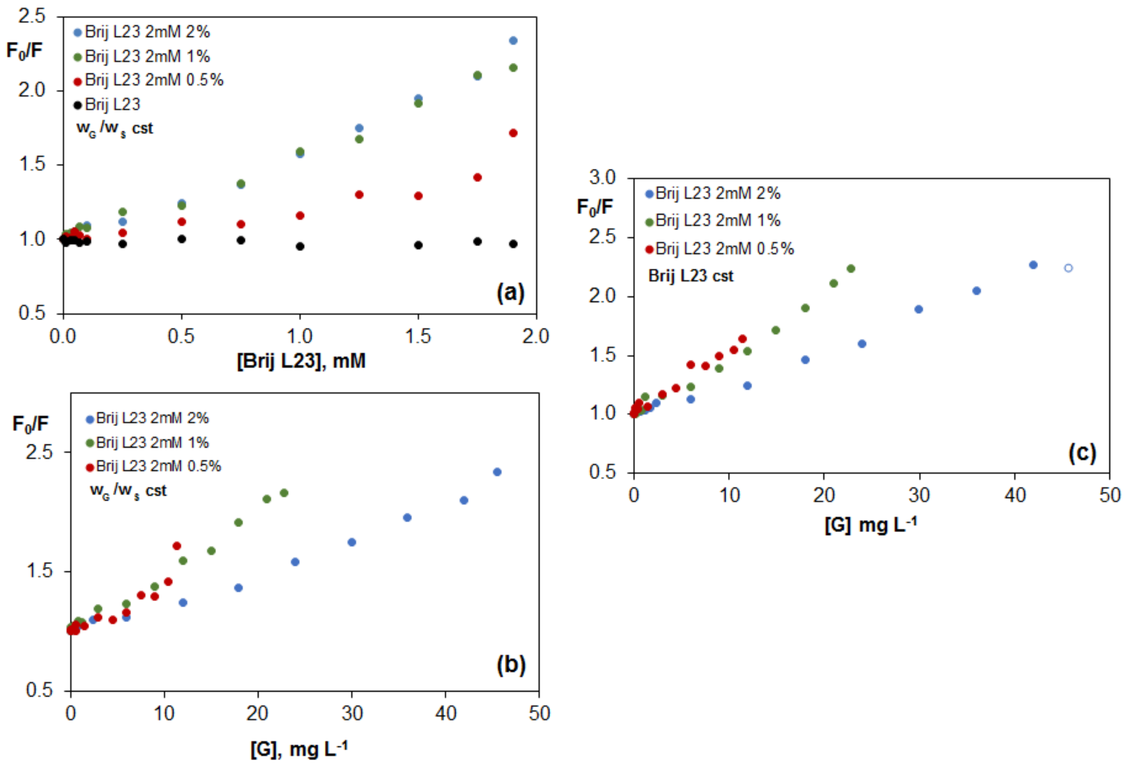

Figure S4. F_0/F for G dispersions in 2 mM Brij L23 as a function of surfactant (a) and G (b and c) concentration, for dispersions with a constant G/surfactant weight ratio (a,b) or constant surfactant concentration (c).

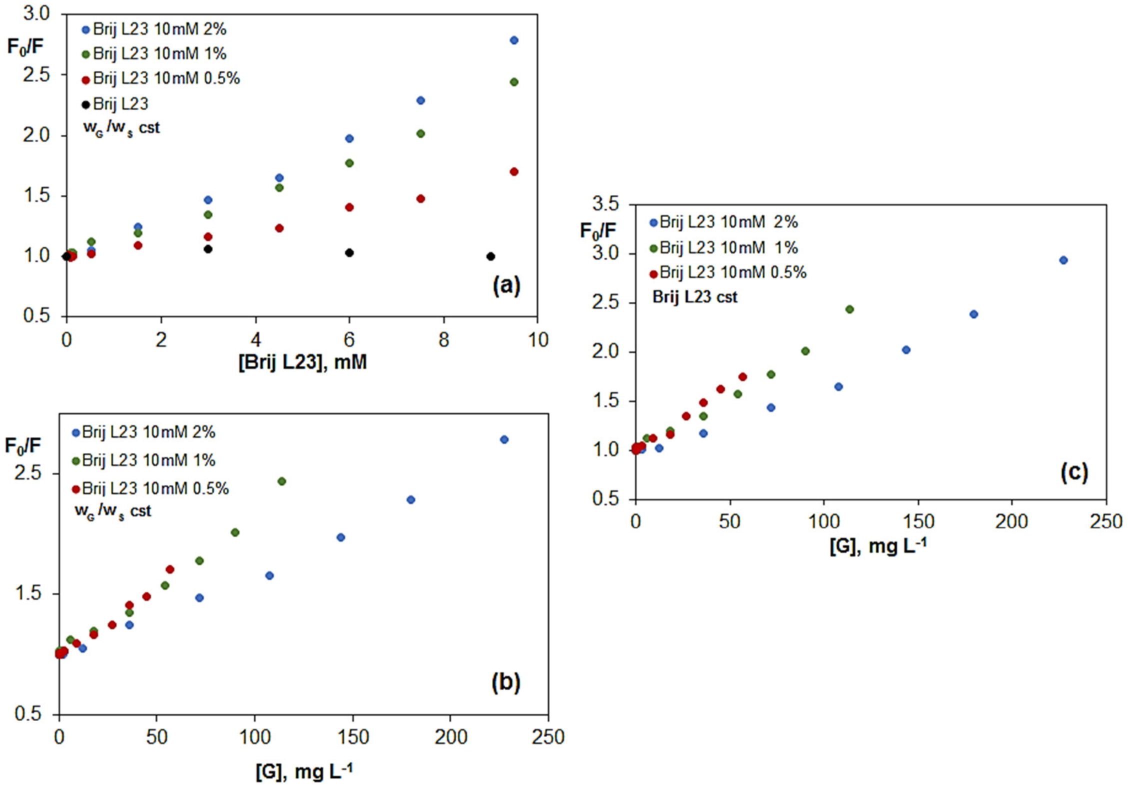

Figure S5. F_0/F for G dispersions in 10 mM Brij L23 as a function of surfactant (a) and G (b and c) concentration, for dispersions with a constant G/surfactant weight ratio (a,b) or constant surfactant concentration (c).

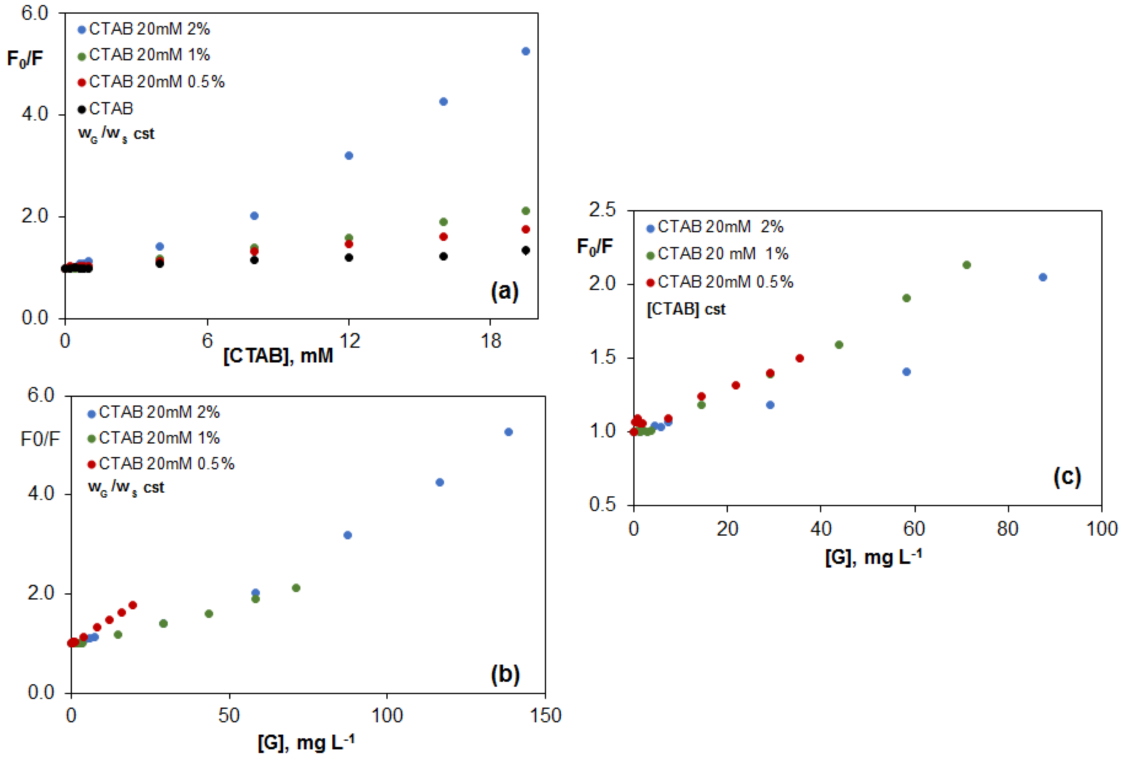

Figure S6. F_0/F for G dispersions in 20 mM CTAB as a function of surfactant (a) and G (b and c) concentration, for dispersions with a constant G/surfactant weight ratio (a,b) or constant surfactant concentration (c).

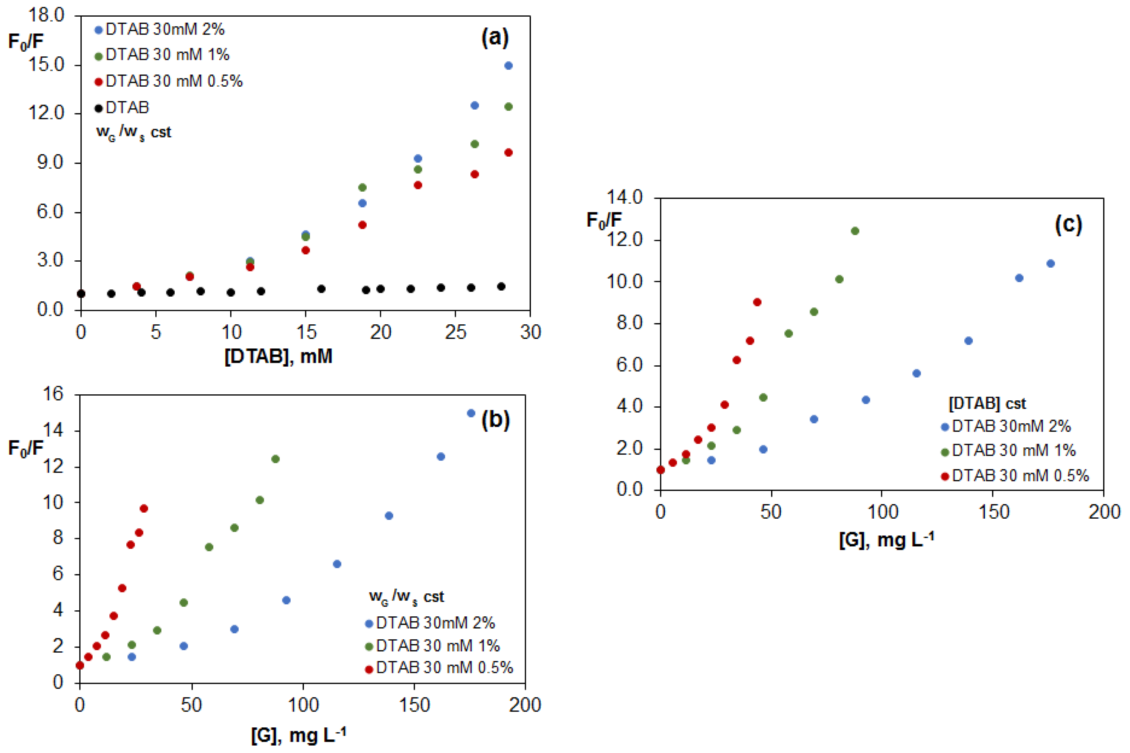

Figure S7. F_0/F for G dispersions in 30 mM DTAB as a function of surfactant (a) and G (b and c) concentration, for dispersions with a constant G/surfactant weight ratio (a,b) or constant surfactant concentration (c).
